# Supplementary material for: Plasma Concentrations of Short-Chain Fatty Acids in Active and Recovered Anorexia Nervosa
Source: Nutrients. 2022 Dec 9;14(24):5247. doi: 10.3390/nu14245247 (PMC9781195; doi:10.3390/nu14245247)
Supplement: Supplementary file 1 [file nutrients-14-05247-s001.zip › Supplementary figure legends.pdf]

Supplementary figure legends:

Supplementary Figure S1. Spearman correlation matrix of SCFA concentrations showing the correlation coefficients. Non-significant correlations (FDR-adjusted $p \geqslant 0.05$) are indicated by a blank.
