# Supplementary figures and images for: Plasma Concentrations of Short-Chain Fatty Acids in Active and Recovered Anorexia Nervosa
Source: Nutrients. 2022 Dec 9;14(24):5247. doi: 10.3390/nu14245247 (PMC9781195; doi:10.3390/nu14245247)

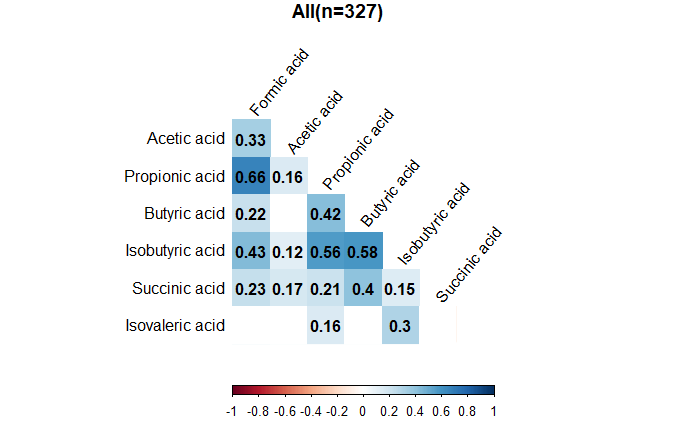

Supplement: Supplementary file 1 [file nutrients-14-05247-s001.zip › Suppl.Figure S1 Xu et al.tif]
